# Correlation between smoking and delirium in patients with sepsis: A retrospective analysis utilizing the MIMIC database

**DOI:** 10.18332/tid/209211

**Published:** 2025-10-16

**Authors:** Renli Wang, Rongjun Liu, Zhaojun Xu, Hua Wang

**Affiliations:** 1 Department of Intensive Care Unit, Ningbo No. 2 Hospital, Ningbo, China; 2 Department of Anesthesiology, Ningbo No. 2 Hospital, Ningbo, China

**Keywords:** sepsis, delirium, smoking, MIMIC-IV database, causal mediation analysis

## Abstract

**INTRODUCTION:**

This study aimed to examine the relationship between smoking and delirium in patients with sepsis and identify potential mediating mechanisms, utilizing data from the Medical Information Mart for Intensive Care-IV (MIMIC-IV) database.

**METHODS:**

A retrospective cohort analysis was conducted involving 10855 adult patients with sepsis. Multivariable logistic regression, propensity score matching (PSM), and inverse probability treatment weighting (IPTW) were applied to assess associations while controlling for confounders such as demographics, comorbidities, vital signs, and laboratory parameters. Causal mediation analysis (CMA) was employed to explore the mediating role of partial pressure of carbon dioxide (PaCO2). Subgroup and sensitivity analyses were performed to assess result robustness.

**RESULTS:**

The incidence of delirium was significantly higher among smokers compared to non-smokers (34.8% vs 25.7%). Adjusted models identified smoking as an independent risk factor (OR=1.44; 95% CI: 1.28–1.61). These findings were validated through PSM (OR=1.35; 95% CI: 1.20–1.53) and IPTW (OR: 1.25, 95% CI: 1.18–1.32). Subgroup analyses affirmed associations across most strata. CMA indicated that 7.876% (95% CI: 4.433–13) of the effect of smoking on delirium was mediated by elevated PaCO2, with direct and indirect effects quantified at 0.0625 (95% CI: 0.0428–0.0800) and 0.0050 (95% CI: 0.0027–0.0081), respectively. Sensitivity analyses among ICU survivors yielded consistent results (OR=1.52; 95% CI: 1.34–1.72).

**CONCLUSIONS:**

Smoking is independently linked to an increased risk of delirium in patients with sepsis, with hypercapnia partially mediating this relationship. These findings emphasize the importance of smoking cessation and targeted respiratory management in preventing delirium.

## INTRODUCTION

Sepsis is a critical condition characterized by organ dysfunction resulting from an aberrant host response to infection^1^. Both sepsis and septic shock represent major healthcare challenges, affecting millions globally each year and causing fatalities in 1 out of 3 to 1 out of 6 affected individuals^2-4^. In the United States, the annual healthcare expenditure related to sepsis exceeds $50 billion, imposing a significant socio-economic burden^5^. Despite advancements in prevention and treatment, sepsis remains inadequately managed due to factors such as an aging population, increasing cancer rates, overuse of immunosuppressants, and antibiotic misuse^6,7^.

Delirium, a common but often undiagnosed neuropsychiatric condition, is characterized by acute fluctuations in consciousness, attention deficits, and cognitive dysfunction^8^. Studies show that 17.7% to 48.0% of ICU patients experience delirium^9^, with its prevalence notably higher among those with sepsis^10^. When delirium occurs in patients with sepsis, it significantly worsens inpatient outcomes, including increased risks of reflux aspiration, falls, weakness, prolonged hospital stays, higher mortality rates, and long-term cognitive impairments^11^. Therefore, it is critical to adopt effective strategies to reduce delirium incidence in patients with sepsis.

Various factors are associated with the development of delirium in patients with sepsis, such as advanced age, alcohol abuse, pre-existing cognitive impairments, infections, surgeries, pain, environmental disturbances, and the use of sedative and analgesic medications^12^. Smoking, a major global health risk, is linked to systemic inflammation, oxidative stress, and immune dysregulation. A large prospective cohort study of 512000 adults in China revealed that smoking increases the risk of 56 diseases, including respiratory, central nervous system, and metabolic disorders, as well as raising the mortality rate for 22 of these conditions^13^. However, the relationship between smoking and delirium remains poorly understood, and there is limited research on this topic, particularly concerning patients with sepsis.

This study leverages the Medical Information Mart for Intensive Care-IV (MIMIC-IV), a robust database providing detailed clinical data on ICU patients. The aim is to investigate the impact of smoking, a modifiable behavioral risk factor, on delirium incidence in patients with sepsis, thereby offering new insights for the early prevention and personalized intervention of delirium in this vulnerable population.

## METHODS

### Database

Data for this study were sourced from the MIMIC-IV database (version 3.0), which contains comprehensive, time-stamped information on over 90000 ICU admissions at Beth Israel Deaconess Medical Center (BIDMC) in Boston, Massachusetts, from 2008 to 2022^14^. The primary author, Renli Wang, completed the Collaborative Institutional Training Initiative (CITI) program and passed the ‘Conflicts of Interest’ and ‘Data or Specimens Only Research’ examinations (Certification ID: 1797679), thereby receiving authorization to access the MIMIC-IV database. The database’s anonymization of patient personal details addresses ethical concerns.

### Study population

**Figure 1:**
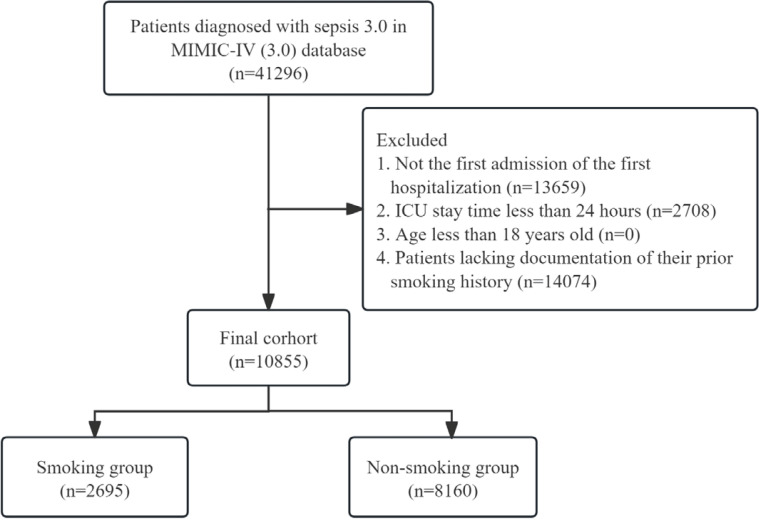
Flowchart depicting the cohort selection procedure, a retrospective analysis, MIMIC-IV database (3.0), 2008–2022 (N=10855)

The inclusion criteria for this study were: 1) a diagnosis of sepsis 3.0; 2) the first ICU admission during the initial hospitalization; 3) an ICU stay exceeding 24 hours; 4) aged >18 years; and 5) documented history of smoking. A flowchart outlining the cohort selection process is provided in Figure 1.

Delirium was assessed in two stages. Initially, the Richmond Agitation and Sedation Scale (RASS) was used for evaluation. Individuals with a RASS score < -3 were classified as being in a coma and did not proceed to the next stage. In the subsequent stage, delirium in eligible patients (RASS score ≥ -3) was assessed using the Confusion Assessment Method for the ICU (CAM-ICU). The CAM-ICU comprises four key features: 1) acute change or fluctuating course of mental status; 2) inattention; 3) disorganized thinking; and 4) altered level of consciousness (LOC). A patient was considered CAM-ICU positive and diagnosed with delirium when both features 1 and 2 were present, along with either feature 3 or 4^15,16^.

### Data extraction and outcomes

Data were extracted using Structured Query Language (SQL) based on PostgreSQL (version 9.6). The following covariates were collected: 1) demographic characteristics such as age, sex, race, alcohol abuse, Charlson comorbidity score, heart failure, severe liver disease, renal disease, chronic pulmonary disease, cerebrovascular disease, malignant cancer, rheumatic disease, diabetes, Sequential Organ Failure Assessment (SOFA) score, Simplified Acute Physiology Score II (SAPS II) score, mechanical ventilation, renal replacement therapy, use of vasoactive drugs, and use of benzodiazepines; 2) initial vital signs upon ICU admission such as heart rate (HR), mean arterial pressure (MAP), respiratory rate (RR), and body temperature (°C); 3) first laboratory test results following ICU admission such as hemoglobin, platelet count, white blood cell (WBC) count, red blood cell (RBC) count, red blood cell distribution width (RDW), prothrombin time (PT), partial thromboplastin time (PPT), blood urea nitrogen (BUN), creatinine, alanine aminotransferase (ALT), aspartate aminotransferase (AST), total bilirubin, albumin, glucose, sodium, potassium, calcium, anion gap, base excess, lactate, partial pressure of oxygen (PaO2), partial pressure of carbon dioxide (PaCO2), and oxygenation index; and 4) smoking history such as individuals with no smoking history or those who have abstained from smoking for over one year were classified as non-smokers, while individuals who are currently smoking or have ceased smoking for less than one year were categorized as smokers. The outcome of this study was the occurrence of delirium.

### Statistical analysis

Variables with missing data exceeding 60% were excluded from the analysis. For variables with less than 60% missing data, multiple imputation using weighted predictive mean matching was performed^17,18^. The *mice* package in RStudio was used for imputation (number of datasets created=20, number of burn in iterations=20)^19^. Supplementary file Table 1 presents the quantity and percentage of missing data for each covariate.

Continuous variables are presented as mean and standard deviation (SD), while categorical variables are reported as frequencies and percentages. Statistical differences between groups for each variable were assessed using the t-test or chi-squared (χ^2^) test.

Based on the smoking criteria mentioned earlier, patients were classified into smoking and non-smoking groups. Logistic regression served as the main analytical approach, with results presented as odds ratios (ORs) and coefficients, accompanied by their respective 95% confidence intervals (95% CIs). Four models were developed to evaluate the effect of smoking on delirium incidence in patients with sepsis: Model 1 unadjusted; Model 2 adjusted for demographic variables; Model 3 adjusted as for Model 2 plus vital signs; and Model 4 adjusted as for Model 3 plus laboratory tests. The variance inflation factor (VIF) was used to detect multicollinearity, and variables with a VIF >4 were excluded. Interaction and stratification analyses were performed considering factors such as age, sex, race, Charlson comorbidity score, SOFA score, mechanical ventilation, and vasopressor use. To ensure robustness, inverse probability of treatment weighting (IPTW) and propensity score matching (PSM) were employed. The effects of PSM and IPTW were assessed before and after matching using standardized mean differences (SMD) and χ^2^ or t-tests. An SMD > 0.1 indicated an imbalance between groups^20^.

To explore whether smoking’s influence on delirium in patients with sepsis is mediated by its effect on respiratory function, causal mediation analysis (CMA) was conducted (using robust standard errors), with changes in PaCO2 as the mediator. CMA decomposes the overall effect of smoking on delirium into direct and indirect effects, referred to as average direct effect (ADE) and average causal mediation effect (ACME), respectively. ADE represents the direct association between smoking and delirium, while ACME represents the indirect effect mediated by PaCO2. This approach provides more comprehensive insights compared to traditional correlation analysis^21^.

Finally, to ensure the stability of the study, only ICU survivors were included in the sensitivity analysis, as death may influence delirium prevalence in a competitive manner.

Statistical analyses were performed using R (version 4.4.2), with a p<0.05 considered statistically significant.

## RESULTS

### Baseline characteristics

A total of 10855 patients diagnosed with sepsis 3.0 were included in this study, with 2695 in the smoking group and 8160 in the non-smoking group. Among the smoking group, 937 individuals (34.8%) developed delirium, whereas 2094 individuals (25.7%) in the non-smoking group experienced delirium. A statistically significant difference was observed between the two groups (p<0.001). Several other characteristics also showed significant differences between the groups. Detailed information is presented in Table 1.

**Table 1:** Baseline characteristics of patients included in the analysis, a retrospective analysis, MIMIC-IV database (3.0), 2008–2022 (N=10855)

*Characteristics*	*All*	*Non-smoking*	*Smoking*	*p*	*SMD*
	*n (%)*	*n (%)*	*n (%)*		
**Total**, n	10855	8160	2695		
**Age** (years), mean (SD)	65.98 (15.98)	68.97 (15.41)	57.72 (14.80)	<0.001	0.727
**Male**	6441 (59.3)	4735 (58.0)	1706 (63.3)	<0.001	0.108
**White**	7234 (66.6)	5525 (67.7)	1709 (63.4)	<0.001	0.090
**Alcohol abuse**	506 (4.7)	191 (2.3)	315 (11.7)	<0.001	0.372
**Comorbidities**					
Charlson score, mean (SD)	5.04 (2.94)	5.28 (2.92)	4.31 (2.90)	<0.001	0.331
Heart failure	3130 (28.8)	2483 (30.4)	647 (24.0)	<0.001	0.145
Severe liver disease	791 (7.3)	478 (5.9)	313 (11.6)	<0.001	0.205
Renal disease	2308 (21.3)	1916 (23.5)	392 (14.5)	<0.001	0.229
Chronic pulmonary disease	2868 (26.4)	1904 (23.3)	964 (35.8)	<0.001	0.275
Cerebrovascular disease	1356 (12.5)	1024 (12.5)	332 (12.3)	0.780	0.007
Malignant cancer	1512 (13.9)	1173 (14.4)	339 (12.6)	0.021	0.053
Rheumatic disease	391 (3.6)	317 (3.9)	74 (2.7)	0.007	0.064
Diabetes	3099 (28.5)	2465 (30.2)	634 (23.5)	<0.001	0.151
**Interventions**					
RRT use	571 (5.3)	415 (5.1)	156 (5.8)	0.172	0.031
MV use	9461 (87.2)	7070 (86.6)	2391 (88.7)	0.006	0.063
Vasoactive drugs use	5425 (50.0)	4111 (50.4)	1314 (48.8)	0.150	0.032
Benzodiazepines use	3643 (33.6)	2669 (32.7)	974 (36.1)	0.001	0.072
	***Mean (SD)***	***Mean (SD)***	***Mean (SD)***		
**Severity**					
SOFA score	5.36 (2.99)	5.29 (2.92)	5.56 (3.16)	<0.001	0.088
SAPS II score	37.86 (13.50)	38.60 (13.40)	35.63 (13.58)	<0.001	0.220
**Vital signs**					
HR (bpm)	89.59 (19.91)	88.98 (19.76)	91.43 (20.25)	<0.001	0.122
MAP (mmHg)	82.16 (18.27)	81.81 (18.11)	83.21 (18.73)	0.001	0.076
RR (bpm)	19.34 (6.18)	19.28 (6.16)	19.50 (6.24)	0.105	0.036
Temperature (°C)	36.69 (0.87)	36.68 (0.86)	36.72 (0.87)	0.018	0.053
**Laboratory tests**					
Hemoglobin (g/dL)	10.53 (2.24)	10.42 (2.17)	10.86 (2.40)	<0.001	0.193
Platelets (×10^9^/L)	195.75 (108.32)	194.37 (106.55)	199.92 (113.43)	0.021	0.050
WBC (×10^9^/L)	13.37 (11.62)	13.34 (12.65)	13.43 (7.68)	0.736	0.008
RBC (×10^12^/L)	3.50 (0.77)	3.48 (0.75)	3.57 (0.831)	<0.001	0.119
RDW (%)	15.02 (2.38)	14.98 (2.34)	15.12 (2.49)	0.008	0.058
PT (s)	16.66 (9.27)	16.88 (9.77)	16.00 (7.50)	<0.001	0.101
PPT (s)	37.57 (22.23)	37.56 (22.09)	37.59 (22.68)	0.938	0.002
BUN (mg/dL)	27.28 (22.10)	28.17 (22.41)	24.58 (20.88)	<0.001	0.166
Creatinine (mg/dL)	1.49 (1.62)	1.52 (1.65)	1.41 (1.52)	0.003	0.068
ALT (u/L)	128.32 (552.18)	108.43 (457.79)	188.52 (767.42)	<0.001	0.127
AST (u/L)	200.86 (995.09)	168.44 (832.81)	299.01 (1369.72)	<0.001	0.115
Total bilirubin (mg/dL)	1.69 (3.78)	1.60 (3.52)	1.99 (4.46)	<0.001	0.097
Albumin (g/dL)	3.05 (0.61)	3.07 (0.61)	3.01 (0.62)	<0.001	0.102
Glucose (mg/dL)	142.64 (69.58)	143.33 (69.65)	140.54 (69.34)	0.070	0.040
Sodium (mmol/L)	137.95 (5.16)	138.05 (5.15)	137.65 (5.18)	0.001	0.077
Potassium (mmol/L)	4.22 (0.73)	4.22 (0.72)	4.12 (0.76)	0.625	0.011
Calcium (mg/dL)	8.21 (0.86)	8.24 (0.86)	8.12 (0.86)	<0.001	0.133
Anion gap (mmol/L)	14.34 (4.35)	14.31 (4.29)	14.42 (4.51)	0.238	0.026
Base excess (mmol/L)	-1.07 (4.89)	-0.98 (4.79)	-1.35 (5.16)	0.001	0.074
Lactate (mmol/L)	2.19 (1.62)	2.21 (1.60)	2.15 (1.65)	0.100	0.036
PaO2 (mmHg)	161.57 (129.64)	164.42 (131.29)	152.95 (124.15)	<0.001	0.090
PaCO2 (mmHg)	41.66 (11.37)	41.01 (10.82)	43.64 (12.67)	<0.001	0.224
Oxygenation index	243.63 (162.30)	246.98 (164.08)	233.48 (156.37)	< 0.001	0.084
**Delirium**	3031 (27.9)	2094 (25.7)	937 (34.8)	<0.001	0.199

SMD: standardized mean differences. RRT: renal replacement therapy. MV: mechanical ventilation. SOFA: Sequential Organ Failure Assessment. SAPS II: Simplified Acute Physiology Score II. HR: heart rate. MAP: mean arterial pressure. RR: respiratory rate. WBC: white blood cell. RBC: red blood cell. RDW: red blood cell distribution width. PT: prothrombin time. PPT: partial thromboplastin time. BUN: blood urea nitrogen. ALT: alanine aminotransferase. AST: aspartate aminotransferase. PaO2: partial pressure of oxygen. PaCO2: partial pressure of carbon dioxide.

### Association between smoking and delirium in patients with sepsis

Table 2 demonstrates that smoking significantly increases the frequency of delirium in patients with sepsis in the unadjusted model (Model 1: OR=1.54; 95% CI: 1.41–1.70, p<0.05), with this association persisting even after adjustment for potential confounders (Model 4: OR=1.44; 95% CI: 1.28–1.61, p<0.05). Confounding bias between groups was minimized through the application of PSM and IPTW methods (Supplementary file: Table 2 and Figure 1). The results remained consistent with PSM (OR=1.35; 95% CI: 1.20–1.53, p<0.05) and IPTW (OR=1.25; 95% CI: 1.18–1.32, p<0.05).

**Table 2:** Association between smoking and delirium in patients with sepsis, a retrospective analysis, MIMIC-IV database (3.0), 2008–2022 (N=10855)

*Model*		*Number of patients*	*OR (95% CI)*	*p*
Unmatched	Model 1	10855	1.54 (1.41–1.70)	<0.05
	Model 2		1.47 (1.32–1.64)	<0.05
	Model 3		1.51 (1.36–1.69)	<0.05
	Model 4		1.44 (1.28–1.61)	<0.05
PSM		4890	1.35 (1.20–1.53)	<0.05
IPTW		21407.8*	1.25 (1.18–1.32)	<0.05

* Weighted pseudo-sample sizes. PSM: propensity score matching. IPTW: inverse probability treatment weighting. Model 1: unadjusted. Model 2: adjusted for demographic variables. Model 3: adjusted as for Model 2 plus vital signs. Model 4: adjusted as for Model 3 plus laboratory tests.

### Subgroup analysis

**Figure 2:**
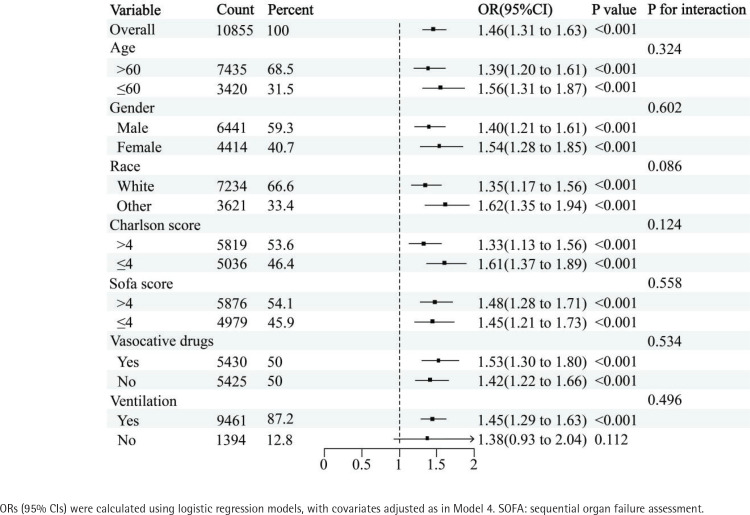
Association between smoking and delirium in different subgroups of patients with sepsis, a retrospective analysis, MIMIC-IV database (3.0), 2008–2022 (N=10855)

Subgroup analysis reveals that, except for the non-mechanical ventilation subgroup (OR=1.38; 95% CI: 0.93–2.04, p=0.112), smoking acts as an independent risk factor for delirium in patients with sepsis across all other subgroups. No significant interactions were observed in any strata (Figure 2).

### Causal mediation analysis

**Figure 3:**
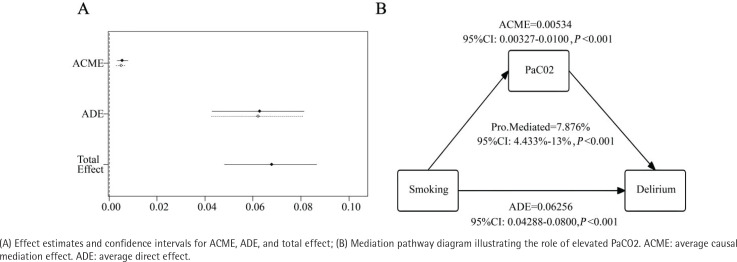
Elevated PaCO2 mediates the association between smoking and delirium, a retrospective analysis, MIMIC-IV database (3.0), 2008–2022 (N=10855)

The CMA analysis (Figure 3) revealed a significant association between smoking and delirium incidence in patients with sepsis. The direct effect was 0.0625 (95% CI: 0.04288–0.0800; p<0.001), while the indirect effect was 0.0050 (95% CI: 0.0027–0.0081). Additionally, 7.876% (95% CI: 4.433–13, p<0.001) of the smoking-related impact on delirium incidence in patients with sepsis is mediated by elevated PaCO2 levels.

### Sensitivity analysis

In the sensitivity analysis focusing solely on patients with sepsis who survived in the ICU, smoking remained an independent risk factor for delirium in sepsis survivors. This association persisted even after adjustment for potential confounders through logistic regression (Model 1: OR=1.64; 95% CI: 1.48–1.82, p<0.05; Model 4: OR=1.52; 95% CI: 1.34–1.72, p<0.05), PSM (OR=1.38; 95% CI: 1.21–1.58, p<0.05), and IPTW (OR=1.31; 95% CI: 1.23–1.40, p<0.05) (Table 3).

**Table 3:** Association between smoking and delirium in patients with sepsis who survived in the ICU, a retrospective analysis, MIMIC-IV database (3.0), 2008–2022 (N=10855)

*Model*		*Number of patients*	*OR (95% CI)*	*p*
Unmatched	Model 1	9464	1.64 (1.48–1.82)	<0.05
	Model 2		1.54 (1.37–1.73)	<0.05
	Model 3		1.58 (1.40–1.78)	<0.05
	Model 4		1.52 (1.34–1.72)	<0.05
PSM		4246	1.38 (1.21–1.58)	<0.05
IPTW		18695.9*	1.31 (1.23–1.40)	<0.05

* Weighted pseudo-sample sizes. PSM: propensity score matching. IPTW: inverse probability treatment weighting. Model 1: unadjusted. Model 2: adjusted for demographic variables. Model 3: adjusted as for Model 2 plus vital signs. Model 4: adjusted as for Model 3 plus laboratory tests.

## DISCUSSION

This retrospective cohort study, leveraging data from the MIMIC-IV database, provides compelling evidence linking smoking to an increased risk of delirium in critically ill patients with sepsis. The results reveal that smokers with sepsis had a 34.8% incidence of delirium, compared to 25.7% in non-smokers, with adjusted models consistently identifying smoking as an independent risk factor. Additionally, causal mediation analysis demonstrated that elevated PaCO2 levels mediated approximately 7.9% of the total effect of smoking on delirium incidence. Hypercapnia, a characteristic of respiratory dysfunction in smokers, impairs cerebral autoregulation, decreases oxygen delivery, and induces metabolic acidosis, all of which may contribute to delirium. These findings have significant implications for delirium prevention in sepsis. First, smoking cessation interventions should be prioritized for high-risk populations, particularly among patients with pre-existing tobacco use. Second, clinicians should exercise increased vigilance for delirium in smokers admitted with sepsis, employing validated screening tools such as the CAM-ICU to facilitate early detection and intervention. Third, optimizing respiratory support to alleviate hypercapnia may help reduce delirium risk in this subgroup. For example, tailored ventilation strategies or targeted management of COPD exacerbations could improve patient outcomes. Finally, these results highlight the need for multidisciplinary care models that integrate addiction medicine, critical care, and neurology to address the complex challenges smoking presents in critically ill patients.

Few studies have explored the correlation between smoking and delirium. Rompaey et al.^22^ analyzed 539 ICU patients and found that the risk of delirium in former smokers was 2.04 times higher than in non-smokers. Similarly, Mardani et al.^23^ investigated 196 patients and identified smoking as an independent risk factor for delirium after coronary artery bypass surgery. However, several studies have reported no association between smoking and delirium. Yoshimura et al.^24^ assessed 100 patients post-liver cancer surgery and concluded that smoking did not increase the risk of delirium in these individuals. Zakriya et al.^25^ surveyed 168 perioperative patients and found no evidence to suggest smoking elevated the incidence of perioperative delirium. These studies are limited by small sample sizes, insufficient adjustment for confounders, and a focus on non-septic individuals. Consequently, the relationship between smoking and delirium in patients with sepsis remains unclear. In contrast, our study provides novel and compelling insights into this issue. The advantages of our research over previous studies include: 1) delirium is relatively common in patients with sepsis, with a complex etiology. Moreover, delirium significantly worsens prognosis in septic individuals, making it clinically important to focus research on this population; 2) the MIMIC database provided a large sample size, making this study, to our knowledge, the largest investigation of the link between smoking and delirium, along with high-quality ICU data, thereby enhancing the reliability of our study; 3) the robustness of our findings was further reinforced by adjusting for potential confounding factors using multivariate logistic regression, PSM, IPTW, subgroup analysis, and sensitivity analysis; and 4) causal mediation analysis offered new insights into the mechanism by which smoking increases the likelihood of delirium in patients with sepsis.

The impact of smoking on the body is highly complex. Although conventional wisdom holds that it is detrimental and has been linked to the development of numerous diseases, recent studies have suggested that its effects on the body may be dual^26^. For example, research indicates that nicotine in tobacco may exert a protective effect against the decline in motor function associated with aging^27^. Although the precise mechanisms by which smoking increases the risk of delirium in patients with sepsis remain unclear, several hypotheses may explain this association. First, smoking induces persistent low-grade inflammation by increasing pro-inflammatory factors such as TNF-α, IL-6, and IL-1β. In sepsis, the ‘cytokine storm’ triggered by infection interacts with the inflammatory effects of smoking, exacerbating blood-brain barrier disruption and central nervous system inflammation, leading to glial cell activation and neuronal damage^28^. Second, free radicals in tobacco, including reactive oxygen species and nitrogen compounds, reduce endogenous antioxidants like glutathione, resulting in oxidative damage accumulation. Mitochondrial dysfunction in sepsis further intensifies oxidative stress, impairs cerebral energy metabolism and DNA repair, and contributes to delirium^29^. Third, smoking induces endothelial dysfunction (e.g. reduced nitric oxide availability, increased endothelin-1 production), which exacerbates septic-related disseminated intravascular coagulation (DIC) and microthrombosis. Abnormal cerebral microcirculation leads to local ischemia and hypoxia, promoting delirium^30^. Fourth, nicotine continuously stimulates nicotinic acetylcholine receptors (nAChR), leading to receptor desensitization. This worsens the impairment of the cholinergic anti-inflammatory pathway during sepsis, amplifying inflammation and triggering delirium^31^. Fifth, smoking influences neuroinflammation-related genes, such as APOE ε4 alleles, through epigenetic mechanisms like DNA methylation and histone modification, increasing vulnerability to delirium^32,33^. Sixth, smoking weakens both innate immunity (e.g. alveolar macrophage function) and adaptive immunity (e.g. T cell responsiveness), heightening the risk of secondary infections. Recurrent infections aggravate the pathophysiological progression of sepsis, indirectly increasing the likelihood of delirium^34^.

Subgroup analysis revealed that smoking did not increase the risk of delirium in patients with sepsis within the non-mechanical ventilation category. The reasons for this divergence remain unclear, but several factors may contribute: 1) the prevalence of delirium in patients with sepsis is influenced by multiple factors, with disease severity and comorbidities being the most prominent. Non-mechanical ventilation patients often experience milder forms of sepsis and have fewer comorbid conditions, resulting in a lower baseline risk of delirium. In such individuals, a history of smoking alone may not be sufficient to trigger delirium; and 2) the relatively small sample size of non-mechanical ventilation patients with sepsis may affect the reliability of these findings.

### Limitations

Several limitations should be acknowledged. First, the MIMIC-IV database primarily includes data from a single academic medical center, potentially limiting the generalizability of the findings to other settings. Second, the retrospective nature of the study inherently limits causal inference, and residual confounding from unmeasured variables (e.g. socioeconomic status, smoking duration, quantity, or secondhand smoke exposure) may persist despite rigorous adjustments. Third, smoking status was dichotomized based on self-reported or documented history, which may underestimate actual exposure levels or fail to capture recent changes in behavior. Fourth, due to the complex nature of delirium diagnosis, some patients may have been overlooked or misdiagnosed (cases potentially confounded with encephalitis or structural brain lesions). Fifth, although mediation analysis provided mechanistic insights, the observational nature of the data prevents definitive conclusions regarding causality in the pathway between smoking, PaCO2, and delirium.

## CONCLUSIONS

This study identified smoking as a significant, modifiable risk factor for delirium in patients with sepsis, with partial mediation through hypercapnia. These findings underscore the importance of addressing tobacco use in sepsis prevention strategies and highlight the need for targeted interventions to reduce delirium risk in this vulnerable population.

## Supplementary Material



## Data Availability

The data supporting this research are available from the following source: https://mimic.mit.edu
