# Supplementary material for: Correlation between smoking and delirium in patients with sepsis: A retrospective analysis utilizing the MIMIC database
Source: Tob Induc Dis. 2025 Oct 16;23:10.18332/tid/209211. doi: 10.18332/tid/209211 (PMC12531987; doi:10.18332/tid/209211)
Supplement: Supplementary file 1 [file TID-23-155-s1.pdf]

Supplementary Table 1. The details on absent data in the pertinent variables, a retrospective analysis, MIMIC-IV database (3.0) , 2008-2022 (N=10855)

Variables	Missing, n (%)
Age	0 (0)
Male	0 (0)
White	0 (0)
Alcohol abuse	0 (0)
Comorbidities	
Charlson score	0 (0)
Heart failure	0 (0)
Severe liver disease	0 (0)
Renal disease	0 (0)
Chronic pulmonary disease	0 (0)
Cerebrovascular disease	0 (0)
Malignant cancer	0 (0)
Rheumatic disease	0 (0)
Diabetes	0 (0)
Interventions	
RRT use	0 (0)
MV use	0 (0)
Vasoactive drugs use	0 (0)
Benzodiazepines use	0 (0)
Severity	
SOFA score	0 (0)
SAPS II score	0 (0)
Vital signs	
HR	0 (0)
MAP	41 (0.377)
RR	0 (0)
Temperature	0 (0)
Laboratory tests	
Hemoglobin	24 (0.221)
Platelets	23 (0.212)
WBC	22 (0.203)
RBC	33 (0.304)
RDW	26 (0.240)
PT	622 (5.730)
PPT	644 (5.932)
BUN	19 (0.175)
Creatinine	20 (0.184)
ALT	4143 (38.167)
AST	4121 (37.964)
Bilirubin total	4156 (38.286)
Albumin	5596 (51.552)

Glucose	46 (0.424)
Sodium	19 (0.175)
Potassium	23 (0.212)
Calcium	695 (6.402)
Anion gap	34 (0.313)
Base excess	2260 (20.820)
Lactate	2904 (26.753)
PaO2	2259 (20.811)
PaCO2	2260 (20.820)
Oxygenation index	4045 (37.264)

RRT, renal replacement therapy; MV, mechanical ventilation; SOFA, Sequential Organ Failure Assessment; SAPS II, Simplified Acute Physiology Score II; HR, heart rate; MAP, mean arterial pressure; RR, respiratory rate; WBC, white blood cell; RBC, red blood cell; RDW, red blood cell distribution width; PT, prothrombin time; PPT, partial thromboplastin time; BUN, blood urea nitrogen; ALT, alamine aminotransferase; AST, aspartate aminotransferase; PaO2, partial pressure of oxygen; PaCO2, partial pressure of carbon dioxide.

Supplementary Table 2. Baseline characteristics and SMD of included patients after PSM and IPTW, a retrospective analysis, MIMIC-IV database (3.0) , 2008-2022 (N=10855)

**Variables**	**PSM**				**IPTW**			
	**Non-smoking**	**Smoking**	***p***	**SMD**	**Non-smoking**	**Smoking**	***p***	**SMD**
N	2245	2445			10998.0	10409.8		
Age (y), mean (SD)	58.35 (16.72)	58.98 (14.28)	0.157	0.040	65.32 (17.14)	64.10 (14.27)	0.006	0.077
Male, n (%)	1543 (63.1)	1536 (62.8)	0.859	0.006	6545.2 (59.5)	6354.3 (61.0)	0.226	0.031
White, n (%)	1562 (63.9)	1561 (63.8)	1.000	0.001	7294.7 (66.3)	6914.4 (66.4)	0.941	0.002
Alcohol abuse , n (%)	170 (7.0)	205 (8.4)	0.068	0.054	548.0 (5.0)	510.4 (4.9)	0.876	0.004
Comorbidities								
Charlson score, mean (SD)	4.31 (3.01)	4.41 (2.92)	0.235	0.034	4.99 (2.99)	4.95 (2.87)	0.685	0.011
Heart failure , n (%)	583 (23.8)	612 (25.0)	0.351	0.028	3111.0 (28.3)	2782.5 (26.7)	0.217	0.035
Severe liver disease , n (%)	260 (10.6)	249 (10.2)	0.640	0.015	836.4 (7.6)	841.4 (8.1)	0.469	0.018
Renal disease, n (%)	382 (15.6)	378 (15.5)	0.906	0.005	2310.2 (21.0)	2344.0 (22.5)	0.237	0.037
Chronic pulmonary disease, n (%)	819 (33.5)	846 (34.6)	0.433	0.023	2948.6 (26.8)	2782.7 (26.7)	0.944	0.002
Cerebrovascular disease , n (%)	309 (12.6)	303 (12.4)	0.829	0.007	1371.7 (12.5)	1306.1 (12.5)	0.936	0.002
Malignant cancer, n (%)	310 (12.7)	321 (13.1)	0.670	0.013	1515.9 (13.8)	1449.4 (13.9)	0.890	0.004
Rheumatic disease , n (%)	70 (2.9)	71 (2.9)	1.000	0.002	393.5 (3.6)	390.6 (3.8)	0.765	0.009
Diabetes , n (%)	586 (24.0)	612 (25.0)	0.406	0.025	3100.1 (28.2)	3006.4 (28.9)	0.598	0.015
Interventions, n (%)								
RRT use	149 (6.1)	132 (5.4)	0.326	0.030	581.5 (5.3)	646.3 (6.2)	0.191	0.040
MV use	2158 (88.3)	2158 (88.3)	1.000	< 0.001	9569.0 (87.0)	9024.9 (86.7)	0.765	0.009
Vasoactive drugs use	1219 (49.9)	1201 (49.1)	0.627	0.015	5481.0 (49.8)	5209.1 (50.0)	0.884	0.004
Benzodiazepines use	887 (36.3)	880 (36.0)	0.858	0.006	3711.6 (33.7)	3558.5 (34.2)	0.740	0.009

Severity, mean (SD)								
SOFA score	5.48 (3.16)	5.49 (3.10)	0.895	0.004	5.39 (3.03)	5.42 (2.99)	0.732	0.009
SAPS II score	35.52 (14.46)	35.79 (13.32)	0.496	0.019	37.77 (13.82)	37.68 (13.12)	0.806	0.007
Vital signs, mean (SD)								
HR (bpm)	91.55 (19.69)	91.19 (20.30)	0.535	0.018	90.02 (20.15)	90.32 (19.97)	0.596	0.015
MAP (mmHg)	82.88 (18.24)	83.12 (18.73)	0.648	0.013	82.19 (18.19)	81.85 (18.84)	0.499	0.019
RR (bpm)	19.45 (6.24)	19.50 (6.31)	0.792	0.008	19.36 (6.20)	19.32 (6.32)	0.805	0.007
Temperature (°C)	36.74 (0.87)	36.73 (0.83)	0.462	0.021	36.70 (0.88)	36.71 (0.82)	0.584	0.014
Laboratory tests, mean (SD)								
Hemoglobin (g/dl)	10.80 (2.23)	10.82 (2.35)	0.770	0.008	10.54 (2.23)	10.45 (2.31)	0.194	0.033
Platelets (× 10^9/l)	201.16 (110.88)	199.90 (111.70)	0.692	0.011	195.81 (108.97)	197.16 (106.86)	0.644	0.013
WBC (× 10^9/l)	13.37 (11.32)	13.43 (7.75)	0.828	0.006	13.43 (12.97)	13.78 (9.88)	0.442	0.031
RBC (× $10^{12/}$l)	3.56 (0.78)	3.57 (0.80)	0.799	0.007	3.50 (0.77)	3.48 (0.80)	0.385	0.025
RDW (%)	15.03 (2.56)	15.07 (2.40)	0.656	0.013	15.01 (2.43)	15.07 (2.32)	0.362	0.025
PT (s)	18.83 (6.09)	15.99 (7.53)	0.419	0.023	16.65 (9.05)	16.44 (8.40)	0.428	0.023
PPT (s)	37.59 (22.74)	37.48 (22.50)	0.859	0.005	37.56 (22.33)	36.88 (20.98)	0.219	0.032
BUN (mg/dl)	24.74 (20.20)	24.81 (20.96)	0.909	0.003	27.16 (21.92)	27.78 (23.80)	0.442	0.027
Creatinine (mg/dl)	1.44 (1.58)	1.41 (1.54)	0.520	0.018	1.49 (1.62)	1.57 (1.72)	0.237	0.043
ALT (u/l)	161.27 (708.50)	160.66 (625.29)	0.975	0.001	132.62 (625.06)	136.49 (553.61)	0.788	0.007
AST (u/l)	257.07 (1225.21)	258.48 (1185.75)	0.967	0.001	203.71 (1018.30)	207.57 (981.34)	0.868	0.004
Total bilirubin (mg/dl)	1.85 (3.88)	1.88 (4.27)	0.836	0.006	1.73 (3.76)	1.83 (4.22)	0.376	0.026
Albumin (g/dl)	3.01 (0.62)	3.01 (0.62)	0.937	0.002	3.05 (0.61)	3.05 (0.61)	0.793	0.007
Glucose (mg/dl)	141.75 (62.80)	140.81 (69.30)	0.621	0.014	142.79 (68.32)	143.34 (76.58)	0.821	0.008
Sodium (mmol/l)	137.79 (5.32)	137.81 (5.02)	0.930	0.003	137.91 (5.26)	137.88 (4.88)	0.790	0.007
Potassium (mmol/l)	4.21 (0.74)	4.21 (0.76)	0.954	0.002	4.22 (0.73)	4.25 (0.77)	0.192	0.040
Calcium (mg/dl)	8.15 (0.84)	8.14 (0.88)	0.591	0.015	8.20 (0.87)	8.20 (0.89)	0.956	0.003
Anion gap (mmol/l)	14.38 (4.28)	14.32 (4.48)	0.613	0.014	14.37 (4.34)	14.38 (4.58)	0.908	0.003
Base excess (mmol/l)	-1.25 (4.81)	-1.27 (5.08)	0.870	0.005	-1.12 (4.84)	-1.23 (5.22)	0.475	0.022
Lactate (mmol/l)	2.15 (1.51)	2.14 (1.65)	0.818	0.007	2.20 (1.59)	2.21 (1.74)	0.815	0.008
PaO2 (mmHg)	154.48 (123.15)	155.35 (125.00)	0.807	0.007	160.98 (129.60)	164.30 (129.41)	0.371	0.026
PaCO2 (mmHg)	43.19 (12.81)	43.35 (12.18)	0.667	0.012	41.91 (12.82)	41.53 (11.09)	0.268	0.032
Oxygenation index	233.50 (151.68)	235.52 (156.86)	0.647	0.013	244.05 (162.05)	247.53 (166.43)	0481	0.021

PSM, Propensity score matching; IPTW, inverse probability of treatment weighing; SMD, standardized mean differences; RRT, renal replacement therapy; MV, mechanical ventilation; SOFA, Sequential Organ Failure Assessment; SAPS II, Simplified Acute Physiology Score II; HR, heart rate; MAP, mean

arterial pressure; RR, respiratory rate; WBC, white blood cell; RBC, red blood cell; RDW, red blood cell distribution width; PT, prothrombin time; PPT, partial thromboplastin time; BUN, blood urea nitrogen; ALT, alamine aminotransferase; AST, aspartate aminotransferase; PaO2, partial pressure of oxygen; PaCO2, partial pressure of carbon dioxide.

Supplementary Figure 1. Love plot of balance in baseline characteristics before and after PSM and IPTW of the patients admitted to the ICU included in the analysis, a retrospective analysis, MIMIC-IV database (3.0) , 2008-2022 (N=10855)

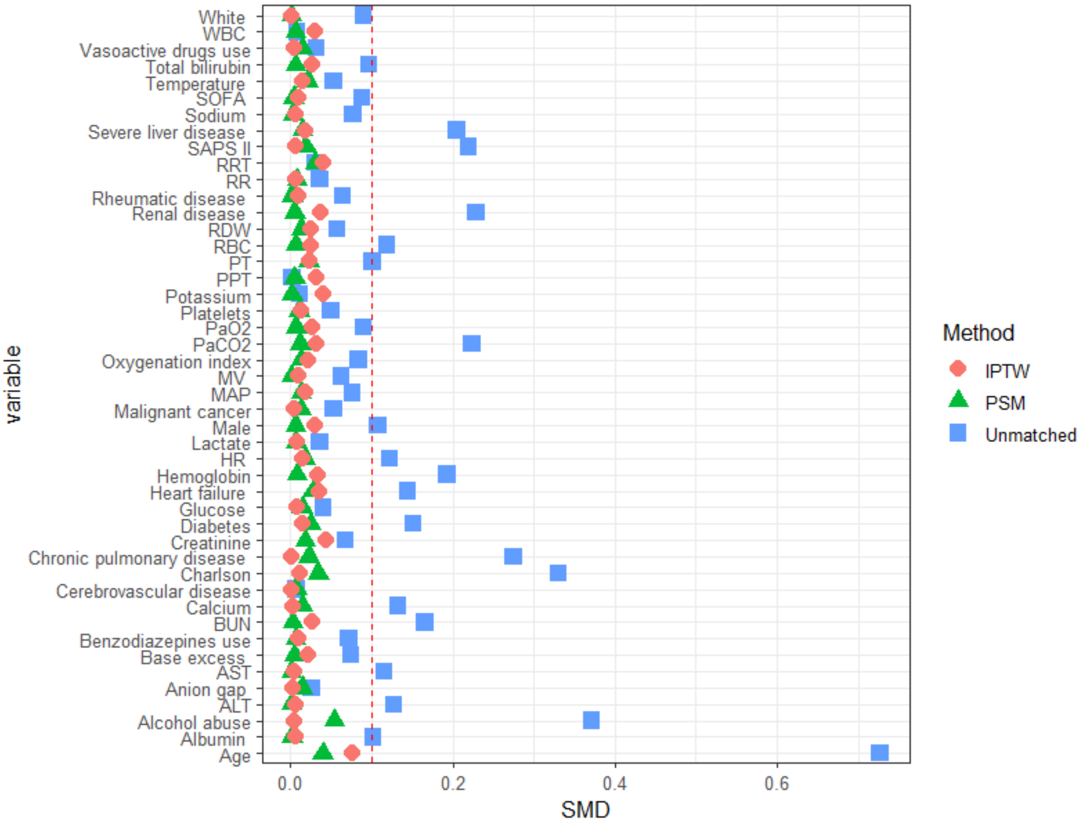

© 2025 Wang R. et al.
